# Lactose-assimilating yeasts with high fatty acid accumulation uncovered by untargeted bioprospecting

**DOI:** 10.1128/aem.01615-24

**Published:** 2024-12-31

**Authors:** Karl Persson, Vanessa O. Onyema, Ijeoma Princess Nwafor, Kameshwara V. R. Peri, Chika Otti, Priscilla Nnaemeka, Chioma Onyishi, Sylvia Okoye, Anene Moneke, Onyetugo Amadi, Jonas Warringer, Cecilia Geijer

**Affiliations:** 1 Department of Life Sciences, Chalmers University of Technology 684649 , Gothenburg, Västra Götaland County, Sweden; 2 Department of Chemistry and Molecular Biology, Gothenburg University 191648 , Gothenburg, Västra Götaland County, Sweden; 3 Department of Microbiology, University of Nigeria 107769 , Nsukka, Enugu, Nigeria; Royal Botanic Gardens, Surrey, United Kingdom

**Keywords:** high-throughput screening, phenotyping, cheese whey, oleaginous yeasts, non-conventional yeasts

## Abstract

Bioprospecting can uncover new yeast strains and species with interesting ecological characteristics and valuable biotechnological traits, such as the capacity to convert different carbon sources from industrial side and waste streams into bioproducts. In this study, we conducted untargeted yeast bioprospecting in tropical West Africa, collecting 1,996 isolates and determining their growth in 70 different environments. While the collection contains numerous isolates with the potential to assimilate several cost-effective and sustainable carbon and nitrogen sources, we focused on characterizing the 203 strains capable of growing on lactose, the main carbon source in the abundant side stream cheese whey from dairy industries. Through internal transcribed spacer sequencing of the lactose-assimilating strains, we identified 30 different yeast species from both the *Ascomycota* and *Basidiomycota* phyla, of which several have not previously been shown to grow on lactose, and some are candidates for new species. Observed differences in growth and ratios of intra- and extracellular lactase activities suggest that the yeasts use a range of different strategies to metabolize lactose. Notably, several basidiomycetous yeasts, including *Apiotrichum mycotoxinivorans*, *Papiliotrema laurentii*, and *Moesziomyces antarcticus*, accumulated lipids up to 40% of their cell dry weight, proving that they can convert lactose into a bioproduct of significant biotechnology interest.

**IMPORTANCE:**

This study paves the way to a better understanding of the natural yeast biodiversity in the largely under-sampled biodiversity hotspot area of tropical West Africa. Our discovery of several yeasts capable of efficiently converting lactose into lipids underscores the value of bioprospecting to identify yeast strains with significant biotechnological potential, which can aid the transition to a circular bioeconomy. Furthermore, the extensive strain collection gathered will facilitate future screening and the development of new cell factories.

## INTRODUCTION

Yeasts have emerged as key players in biotechnology and molecular and evolutionary biology (1–3). Tens to hundreds of thousands of yeast species are expected to exist in nature, of which only a tiny fraction (<3,000) has been identified so far, and only a handful of these have been extensively characterized (4). By identifying and characterizing more yeast species and strains, we can broaden our understanding of yeast biodiversity and their impact on nature. In addition, the natural biodiversity of yeasts represents a largely untapped resource for industrial applications, where the yeasts can be used as cell factories to produce fermented foods and beverages and synthesize proteins, lipids, and other metabolites of industrial interest (5).

Yeasts exist in almost all known natural biomes and are particularly prevalent on the surface of, or within, fruits, plant exudates, soil, insects, rotting wood, and tree bark (6). Yeasts also interact with humans as beneficial agents in fermentation and as commensal microbes in the human microbiota. In some cases, they may act as opportunistic pathogens, especially threatening individuals with weakened immune systems (7). Whereas the yeast biodiversity of North America, Europe, and parts of Asia is somewhat well mapped, plenty of niches and geographical locations in the global south remain only superficially explored. These include biodiversity hotspots in tropical Africa and South America that likely hold many new yeast species and lineages with ecologically or industrially interesting traits (4, 8).

Novel yeast strains and species are often found through bioprospecting—the systematic and organized search for useful products derived from bioresources (9). Untargeted bioprospecting aims to collect every microbe that grows on non-selective plates in the laboratory, while targeted bioprospecting gathers microbes that grow well in a select environment, often of industrial interest. Robotics and high-throughput screening have speeded up key steps in bioprospecting, but creating a genetically diverse strain collection is still time-consuming and labor-intensive. Beyond strain isolation, bioprospecting requires pure-streaking of colonies to obtain monocultures and DNA-sequencing of isolates to determine their taxonomic position. To achieve the latter, genomic regions that vary extensively between isolates, but that are flanked by conserved sequences, such as the internal transcribed spacer (ITS) region or the D1/D2 domain of rDNA, are often sequenced by targeted short read sequencing and matched to known species sequences in online databases (10). Once established, the resulting strain collections and screening platforms can be used extensively and repeatedly to screen for different combinations of desired characteristics, and ultimately for identifying yeast cell factories for the transformation of inexpensive and renewable substrates into a spectrum of essential biochemicals (11, 12).

To work as a cell factory, the yeast should be easy to store, maintain, and cultivate and be fast and efficient in converting available substrates to desired products without the requirements of expensive additives in the culture medium (13). Currently, industrial yeast bioconversion processes are most often carried out by domesticated lineages of the baking, brewing, and wine yeast *Saccharomyces cerevisiae* (14). Domesticated *S. cerevisiae* ferments simple six-carbon sugars, such as glucose, exceedingly well, while using a variety of widely available nitrogen, phosphor, and sulfur compounds to support its growth, and needs only a few externally added complex nutrients, such as vitamins. Unfortunately, *S. cerevisiae* cannot naturally grow on many carbon sources that are abundant in industrial side and waste streams, including monosaccharides such as xylose, arabinose, agricultural fructose, disaccharides such as cellobiose and lactose, as well as more complex oligo- and polysaccharides, including starch, cellulose, and hemicelluloses. For example, the inability of *S. cerevisiae* to metabolize lactose restricts its use in valorizing cheese whey, which consists of 4.5–5% lactose, proteins, fatty acids, and minerals. With a global annual production of 100 million tons, whey constitutes a substantial by-product of the dairy industry with significant potential as a raw material for bioprocessing (15). The ability to cleave the β-1,4 glucosidic bond between lactose’s glucose and galactose moieties using lactases (normally β-galactosidases) and assimilate the breakdown products is relatively rare in the fungal kingdom. For example, among a panel of 332 sequenced ascomycetous yeasts, less than 10% could grow on this carbohydrate (16). Among the known lactose-assimilating yeasts, “dairy” yeasts from the *Kluyveromyces* genus have been carefully characterized (17), and *K. lactis* and *K. marxianus* are the predominant species employed in current whey bioconversion processes (18, 19). Other lactose-assimilating yeasts remain poorly explored but may have considerable potential for commercialization by broadening the range of metabolites that can be produced from whey.

One such class of industrially interesting metabolites is lipids, which can be used in society as fuels, chemicals, and food ingredients. Lipids usually make up around 6–8% (wt/wt) of the cell dry weight (20), whereas yeasts that accumulate more than 20% of cell dry weight in lipids are collectively referred to as oleaginous yeasts (21). Oleaginous yeast species such as *Yarrovia lipolytica*, *Rhodotorula toruloides*, and *Cutaneotrichosporon oleaginosus* are highly proficient lipid producers and are currently being characterized and developed into cell factories for the production of lipids and various lipid derivatives. The lipids accumulated in these oleaginous yeasts consist predominantly of triacylglycerols (TAGs), which are stored in cell lipid bodies. TAG production from sugar is typically stimulated when carbon is in excess and another nutrient, such as nitrogen, is growth-limiting (22). Importantly, yeast species and strains differ in the types and amounts of TAGs they produce, and the TAG profiles also depend on the carbon source and cultivation conditions used (23). Thus, identifying and developing new cell factories that efficiently convert carbon sources in abundant industrial side and waste streams into lipids is of great interest.

In this study, we used untargeted bioprospecting to discover evolutionary diverse yeasts in tropical West Africa, one of the most species-rich but, in terms of yeast, least explored regions (8). Our comprehensive collection of 1,996 isolates underwent rigorous high-throughput screening to accurately measure the capacity of each isolate to grow on a broad spectrum of substrates and in conditions capturing the effect of various industrial stress factors. While the collection contained many isolates with a diverse potential of assimilating cheap and sustainable substrates, we particularly focused on characterizing those capable of converting lactose and found several basidiomycetes that could produce high amounts of lipids from this carbon source. Our approach illustrates how unbiased bioprospecting of the tropics can be used to tap its unexplored natural yeast biodiversity to identify isolates with industrially promising properties.

## RESULTS

### Untargeted bioprospecting of Nigerian yeasts

**Fig 1:**
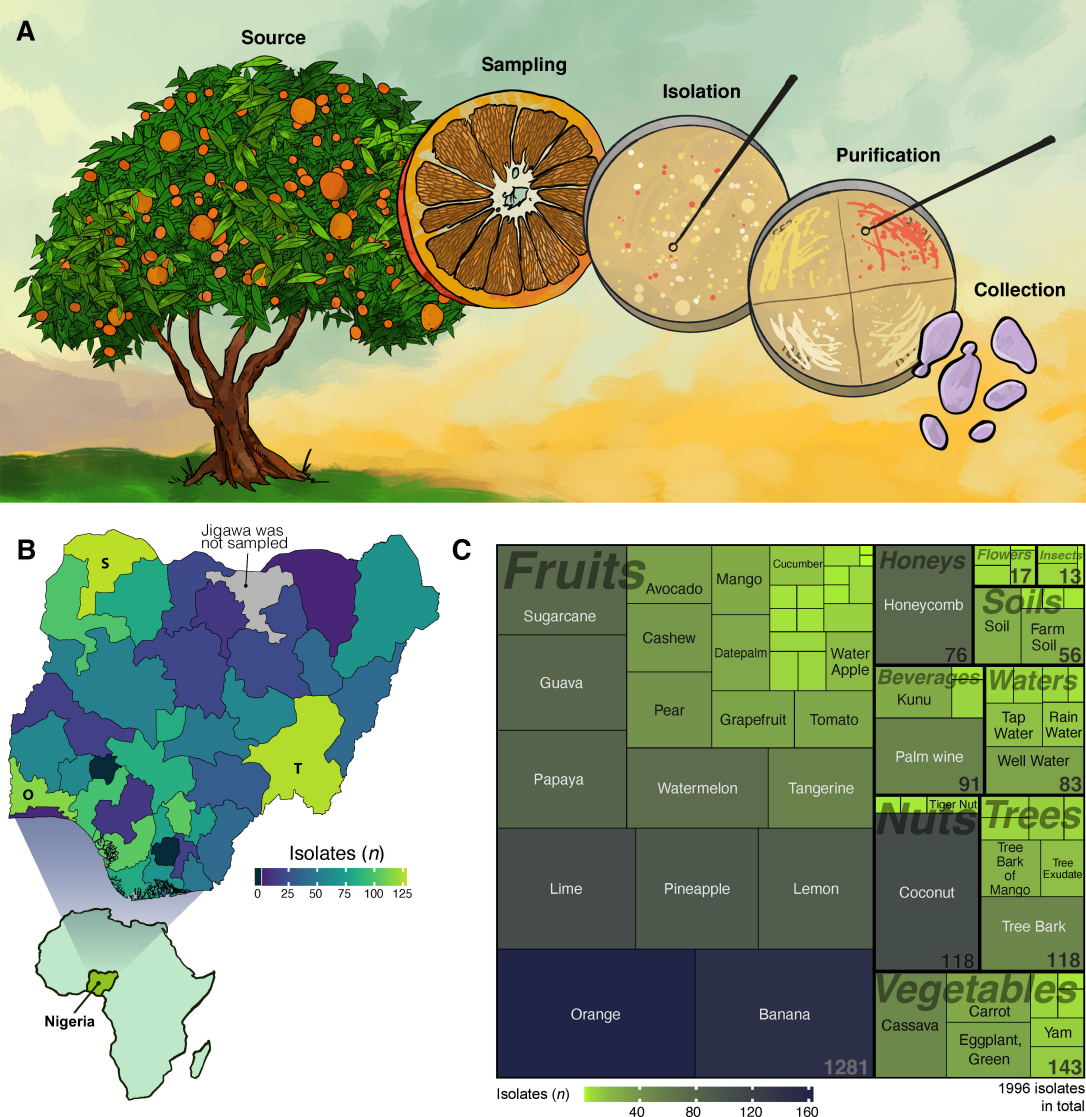
Untargeted bioprospecting in Nigeria. (**A**) Yeast isolation strategy. Samples were collected in sterile plastic bags, transferred to water, and spread on SSEM medium. After 24–72 h of growth, diverse single colonies were pure-streaked, and further selected on chloramphenicol (200 mg/L), ampicillin (100 mg/L), and gentamycin (20 mg/L) to remove potential bacterial contaminants. The final collection was stored in 20% glycerol at −80°C. (**B**) Geographic origin of samples. Nigerian states are color-coded according to the number of isolates in the final strain collection. States Sokoto, Taraba, and Ogun represent the three states with highest number of isolates, and are labeled with S, T, and O, respectively. Jigawa was not sampled. The inset map was generated using GADM (Global Administrative Areas), version 4.1, accessed via the raster::getData() function in R. (**C**) Source material of samples. The tree-map chart shows the proportions and types of source materials from which the strains were isolated. Fields are color-coded by the number of isolates of each source material. Numbers in the bottom right corner show the number of samples from each source-category (e.g., fruits).

To extensively sample a previously underexplored tropical region, we performed the first country-wide yeast bioprospecting of Nigeria, sampling all states except Jigawa, with the highest number of yeast isolates originating from Sokoto, Taraba, and Ogun (number of isolates (*n*) = 128, 126, and 116, respectively) (Fig. 1A and B). We not only collected biological samples from presumed yeast-rich niches, such as fruits and fermented beverages, but also included samples from barks, herbs, soils, and waterways (Fig. 1C). Samples were transferred to agar plates with a cultivation medium formulated to promote yeast growth while inhibiting bacterial growth. From these plates, we selected pure-streaked, smooth, yeast-like colonies. The work resulted in a collection of 1,996 isolates from >300 distinct biological samples. While most isolates are yeasts, we cannot exclude that some bacterial strains that are naturally resistant to a broad range of antibiotics may have bypassed our antibacterial selection process.

### Isolates display diverse growth profiles

**Fig 2:**
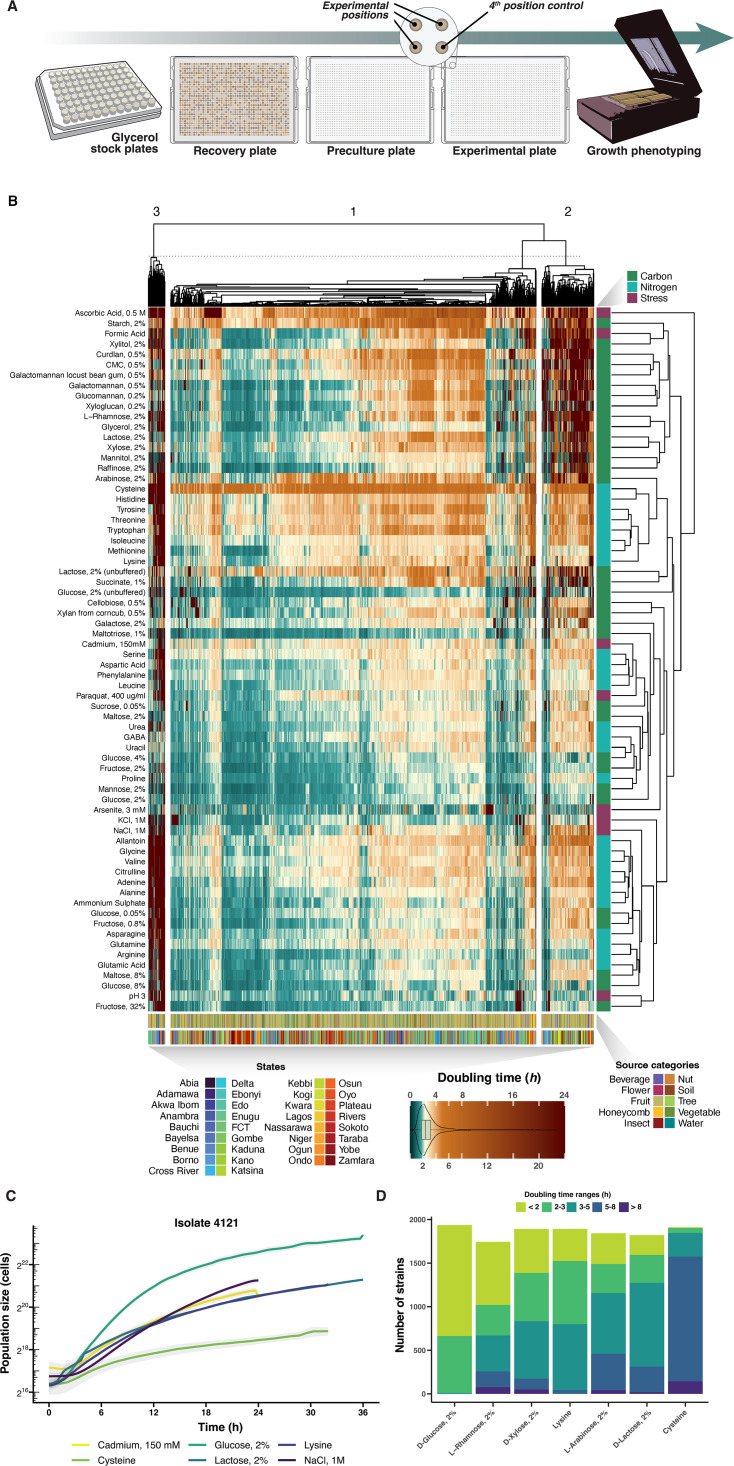
Growth phenotyping of bioprospected strains in high-throughput. (**A**) Design of colony growth experiments on agar medium. Isolates were recovered from glycerol stocks and robotically arranged in 1,536 colony array grids on recovery (YPD) plates and then transferred first to pre-cultivation plates (SC, 2% glucose) and then to experimental plates (SC with different carbon and nitrogen sources as well as stress factors). (**B**) Heatmap showing the cell doubling time of all isolates (*x*-axis, *n* = 1,996) in all tested environments (*y*-axis). Isolates and strains were grouped based on similarity in doubling time profiles using hierarchical clustering (Euclidian distance metric, median used for groups). Numbers to the left indicate major group, with the broken line indicating the delineation of these groups. Figure legends show the color of states, source categories, environmental classes, and doubling time values. The doubling times (hours) are color-coded according to the gradient, while the combined violin and box plot visualizes the dataset’s total distribution. (**C**) Example growth curves of one strain in select environments. The *y*-axis shows cell counts in cell populations. (**D**) Stacked bar plot showing cumulative numbers of yeast isolates with a range of doubling times in glucose and in environments, where wild-type *S. cerevisiae* fails to grow.

To enable high-throughput growth characterization, we recovered frozen isolate stocks in 1,536 colony arrays on agar plates. We transferred the recovered colonies first to preculture plates and then to experimental plates for measurement of population sizes at dense intervals during 2–3 days of growth in 70 environments (Fig. 2A). These environments were chosen to capture the strains’ abilities to use carbon and nitrogen sources and to tolerate stresses, such as high osmolarity and low pH, that can be present during industrial bioprocessing.

We measured population sizes by automated scanning of the light transmitted through each colony, transformed the pixel intensities into cell counts, and extracted the cell doubling times as a proxy for fitness from >700,000 growth curves (Fig. 2B) (24). Strains isolated from the same geographic origin (Nigerian state) or source material (e.g., fruits) did not group into larger clusters (Fig. 2B). This suggests that the source habitats harbor diverse sets of microorganisms and that phenotypically similar species are present across the range of sampled sources and locations.

A hierarchical clustering (Fig. 2B) categorized the isolates into three major growth groups: a fast-growing group of 1,675 strains (83.9%) with generally fast growth across environments, a hexose-preferring group of 239 (12.0%) strains that thrive if supplied with hexoses, but struggle when using pentoses, sugar alcohols, and complex polysaccharides as carbon, and a small, slow-growing group of 77 isolates (3.85%) with mostly poor growth across environments. Thus, in agreement with (25), we see that many isolates consistently performed well, whereas others consistently performed poorly, arguing against the “jack of all trades, master of none” trade-off among yeasts. Figure 2C illustrates growth curves underlying doubling time estimates.

A limitation of the division into three supergroups is that it hides the considerable variation in growth in different environments, including the remarkable number of strains capable of fast growth in some environments. For example, many strains achieved substantial (<8 h doubling time), moderate (<5 h), or even fast (<3 h) growth in environments where *S. cerevisiae* typically struggle, such as on the carbon sources arabinose, lactose, and xylose, and on cysteine and lysine as sole nitrogen sources (Fig. 2D). In fact, in each of these environments, 13.6% of the strains achieved fast growth (<3 h doubling time), excluding cysteine where only 66 strains achieved fast growth. Untargeted bioprospecting of underexplored regions can therefore uncover strains that grow well in many environments of potential industrial interest.

### Identification of yeasts growing on lactose agar medium

Lactose-rich whey is currently an under-utilized industrial side stream that can be valorized through microbial conversion into a range of metabolites of societal interest. That 27.4% of our Nigerian strains achieved fast (<3 h doubling time) lactose growth (Fig. 2D) is thus of particular interest to investigate further, especially given that: (i) our bioprospecting was untargeted and did not include lactose-rich niches, such as dairy farms and products, and (ii) lactose-assimilation is considered a rare trait in yeast (16).

To ensure pure monocultures for further downstream analysis, we streaked the fastest lactose-growing strains on separate synthetic complete (SC) agar plates containing 2% lactose, and isolated colonies expanded from individual cells. Of the ~400 selected lactose-growing strains, 203 strains maintained their lactose growth throughout two to three re-streaking passages. From these, we extracted the total DNA, amplified and sequenced their ITS regions, and matched the resulting ITS sequences with sequenced type yeasts in the NCBI BLAST database. The ITS sequences mapped to 30 genetically distinct lactose-utilizing yeast species from both the *Ascomycota* and *Basidiomycota* phyla (Table 1). A handful of bacterial isolates were also identified and removed from the lactose-assimilating data set, underscoring that the collection is highly enriched for, but does not exclusively contain, yeasts. Although most of the ITS sequences aligned perfectly or nearly perfectly with those of known yeasts, 57 of our lactose-growing isolates exhibited less than the 98.41% identity threshold suggested by Vu et al. to delineate yeast species (26). Of these, 27 isolates exhibited no ITS type-strain match with a similarity higher than 96% to any known species, indicating that they may be candidates for new species (Table 1). However, for simplicity, we call all isolates by the species names of their closest BLAST-hits.

**TABLE 1:** Lactose assimilating strains

Species	Division	Lactose growth (+/−)	Isolates (*n*)	Sources	States (*n*)	Sequence identity (%)
*Apiotrichum mycotoxinivorans*	*Basidiomycota*	−^*a*^	5	Citrus fruits (4), water (1)	5	99.87 ± 0.30
*Candida orthopsilosis*	*Ascomycota*	−^*a*^	1	Coconut	1	99.31
*Candida palmioleophila*	*Ascomycota*	−	5	Banana (2), pineapple (2), orange (1)	1	99.82 ± 0.00
*Candida parapsilosis*	*Ascomycota*	−	1	Coconut	1	100
*Candida pseudocylindracea*	*Ascomycota*	+	1	Water apple	1	95.97
*Candida tropicalis*	*Ascomycota*	+	12	Various fruits (6), various nuts (4), carrot (1), water (1)	6	97.28 ± 2.60
*Cutaneotrichosporon curvatum*	*Basidiomycota*	−	1	Papaya	1	99.78
*Cyberlindnera fabianii*	*Ascomycota*	−	1	Tree bark	1	99.64
*Debaryomyces nepalensis*	*Ascomycota*	+	4	Coconut (1), water (1), lime (1), sugarcane (1)	3	98.48 ± 2.58
*Kodamaea ohmeri*	*Ascomycota*	−^*a*^	2	Bark of orange tree (1), pineapple (1)	2	98.48 ± 0.03
*Kurtzmaniella quercitrusa*	*Ascomycota*	−^*a*^	1	Tangerine	1	96.82
*Meyerozyma caribbica*	*Ascomycota*	−	45	Various fruits (25), various tree barks (6), cucumbers (6), coconuts (3), soils (3), honeycombs (2), tree exudate (1)	13	99.73 ± 0.34
*Meyerozyma carpophila*	*Ascomycota*	−	3	Avocado (1), banana (1), tomato (1)	3	100.00 ± 0.00
*Meyerozyma guilliermondii*	*Ascomycota*	−	3	Bark of orange tree (2), coconut (1)	2	99.39 ± 0.38
*Moesziomyces antarcticus*	*Basidiomycota*	+	1	Lemon	1	96.73
*Papiliotrema flavescens*	*Basidiomycota*	+	2	Soil	1	100.00 ± 0.00
*Papiliotrema laurentii*	*Basidiomycota*	+	2	Guava (2)	1	97.65 ± 0.04
*Papiliotrema rajasthanensis*	*Basidiomycota*	+	1	Eggplant (1)	1	99.75
*Pichia kudriavzevii*	*Ascomycota*	−	5	Citrus fruits (2), coconuts (2), water (1)	4	92.38 ± 5.51
*Rhodosporidiobolus ruineniae*	*Basidiomycota*	−	2	Velvet tamarind (2)	2	98.73 ± 0.74
*Rhodotorula mucilaginosa*	*Basidiomycota*	−	2	Lime (2)	1	96.89 ± 3.35
*Sungouiella intermedia*	*Ascomycota*	+	19	Various fruits (18), water (1)	9	95.75 ± 1.27
*Sungouiella pseudointermedia*	*Ascomycota*	+	57	Various fruits (40), various tree bark (8), cassava (2), soil (2), water (2), coconut (1), caterpillar (1)	15	99.39 ± 1.07
*Trichosporon asahii*	*Basidiomycota*	+	7	Various fruits (4), various vegetables (3)	4	99.64 ± 0.63
*Trichosporon insectorum*	*Basidiomycota*	+	1	Soil	1	100
*Ustilago sparsa*	*Basidiomycota*	n.a.^*b*^	1	Lemon	1	97.89
*Vishniacozyma taibaiensis*	*Basidiomycota*	−	1	Lemon	1	99.78
*Yamadazyma mexicana*	*Ascomycota*	+	1	Cassava	1	99.81
*Yarrowia galli*	*Ascomycota*	−	4	Pineapple (2), avocado (1), coconut (1)	3	97.06 ± 0.89
*Yarrowia lipolytica*	*Ascomycota*	−	10	Various fruits (10), coconut (1)	6	98.58 ± 0.08

^*a*^ Strain(s) shown to grow on lactose, although http://theyeasts.org/ states “no lactose growth.” See Table S1 for information on individual strains and their sources of isolation.

^*b*^ n. a. = not available.

^*c*^ Ability of species to grow on lactose (+) or not (−) as stated by theyeasts.org

More than half (*n* = 18) of the 30 lactose-utilizing yeast species were isolated multiple times, with the most common being *Sungouiella pseudointermedia* (57 isolates, formerly *Candida pseudointermedia*), followed by *Meyerozyma caribbica* (27), *Sungouiella intermedia* (19, formerly *Candida intermedia*), *Candida tropicalis* (12), and *Yarrowia lipolytica* (10). For each of these species, the lactose-assimilating strains were isolated from a wide variety of geographic origins and source materials (Table 1), making a shared recent clonal ancestry unlikely. This suggests that lactose growth is a prevalent property in these species and is not restricted to rare strains from a particular region or habitat. We note that while lactose assimilating isolates of some of these species have been identified before, 17 of our species (marked as - for "Lactose assimilating" in Table 1) are not listed as lactose-utilizing on the http://theyeasts.org/ webpage, which provides the most up-to-date and accurate taxonomic and phenotypic information on published yeasts (4). For the yeasts listed as negative for lactose assimilation, we did a literature search and confirmed that most of these species have indeed not previously been shown to grow on lactose, even if strains belonging to these species had been isolated from dairy products in some cases. However, strains of *Apiotrichum mycotoxinivorans*, *Kodamaea ohmeri*, *Kurtzmaniella quercitrusa*, and *Candida orthopsilosis* have been shown to grow on lactose (28–31) and are therefore marked with −^*a*^ in Table 1. Overall, we conclude that the data set contains a range of evolutionary distinct species, where some have been previously shown to assimilate lactose and others have not, as well as several potentially new species.

### Yeast isolates display robust yeast biomass production in cheese whey

To evaluate whether the 203 isolates capable of growth on SC lactose agar plates could also use liquid whey as a growth substrate, we prepared fresh whey using a cheese-making kit. The whey was filter-sterilized to eliminate contaminating microorganisms and then used as the sole growth substrate for the strains in liquid microscale cultivations, without the addition of any supplementary nutrients.

**Fig 3:**
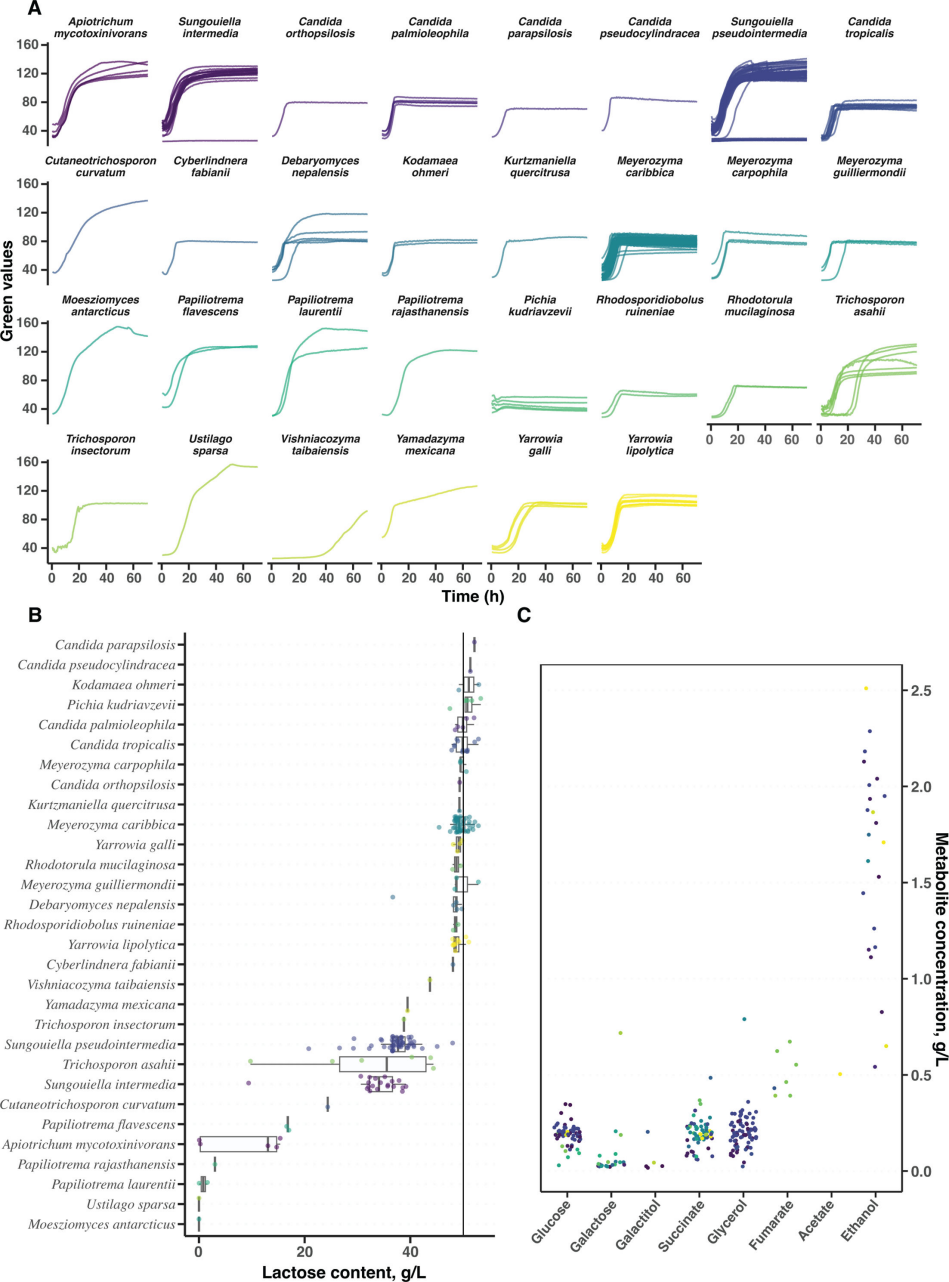
Yeasts growing on lactose in cheese whey. (**A**) Growth curves of strains growing on filter sterilized cheese whey in microscale liquid cultures; samples for HPLC were collected at the end points. (**B**) Lactose left in the supernatant after the cultivation was determined by HPLC. The lateral line indicates the concentration of lactose in whey before inoculation. (**C**) Traces of other metabolites found in whey after cultivation, color according to species, as in (A) and (B).

Almost all isolates (94.6%) exhibited robust growth (mean doubling time of 2.17 h) in whey (Fig. 3A). While lactose is the primary carbon and energy source in cheese whey, it also contains smaller amounts of amino acids, organic acids, and lipids that may serve as alternative carbon sources for the yeasts (32). Whey can also contain traces of glucose and galactose, although high-performance liquid chromatography (HPLC) analysis showed that the levels of glucose and galactose in our in-house produced whey were below detection limits. Thus, to ensure that the recorded growth was accompanied by lactose consumption, we analyzed the lactose content of the whey before and after three days of microscale liquid cultivation (Fig. 3B). We found that especially the basidiomycetous yeasts *M. antarcticus*, *Ustilago sparsa*, *P. laurentii*, and *Papiliotrema rajasthanensis* had consumed all, or almost all, the available lactose. Other yeast species, including the ascomycetes *S. (pseudo)intermedia*, displayed a more moderate consumption, averaging 27.6%. For these species, there was substantial variation in lactose consumption among different strains. However, many species with good growth on whey failed to consume substantial amounts of the lactose available and must thus have achieved their growth primarily by catabolizing other carbon sources present in the whey (Fig. S2A). This may not necessarily reflect an inability to use lactose in whey but could indicate suppression of lactose utilization by the presence of a preferred carbon source, similar to the glucose repression of galactose assimilation in *S. cerevisiae* (33).

To track the fate of the carbon consumed by our lactose-growing yeast isolates, we also measured the extracellular accumulation of the lactose hydrolysis products glucose and galactose, as well as a variety of small carbon compounds that often serve as end products of carbon catabolism. Overall, we found multiple species that produced small but detectable levels of glucose, galactose, galactitol, succinate, and glycerol. *Trichosporon asahii*, *Trichosporon insectorum*, and *Cutaneotrichosporon curvatum* produced approx. 0.5 g/L of fumarate, and moderate amounts (0.5–2.3 g/L) of ethanol were produced by some isolates of *Apiotrichum mycotoxinovorans*, *Candida orthopsilosis*, *Candida palmioleophila*, *S. (pesudo)intermedia*, *Debaryomyces nepalensis*, *M. caribbica*, *Yamadazyma mexicana*, *Yarrowia galli*, and *Yarrowia lipolytica* (Fig. 3C; Fig. S2B). However, in general, the carbon contained in these small carbon compounds was negligible, indicating that most of the carbon consumed had been channeled into yeast biomass and/or carbon dioxide (not measured). Thus, modifications to either the whey composition, the operational conditions or the yeast strains themselves may be necessary to optimize the production of these and other relevant metabolites.

### Yeast isolates differ in their capacities to grow on and hydrolyze lactose

**Fig 4:**
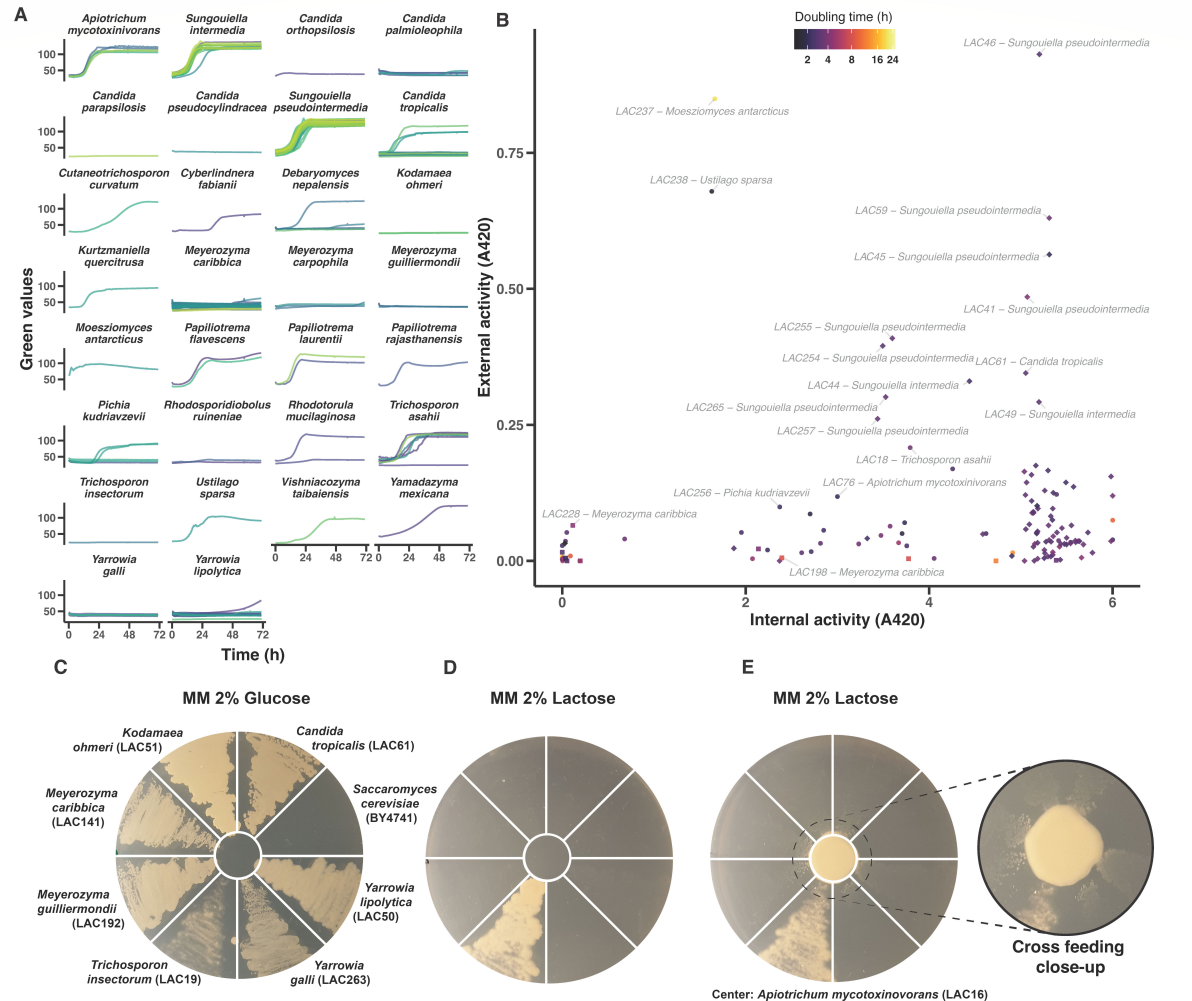
Yeasts growing in liquid and hydrolyzing lactose. (**A**) Growth of yeast isolates in microscale liquid MM 2% lactose medium. (**B**) β-galactosidase activity measured in cells (*x*-axis) and supernatant (*y*-axis) after final time points in (A). Point shapes are round for all genus, except for *Candida/Sungouiella* (diamonds) and *Meyerozyma* (squares). (**C**) Strains streaked on MM glucose plates with the negative control ScBY4741 in the center. (**D**) Strains are in the same layout as in (C), streaked on MM lactose plates with the negative control ScBY4741 in the center. (**E**) Strains in the same layout as in (C) and (D), streaked on MM lactose plates with *A. mycotoxinivorans* streaked in the center; close-up shows a zone of growth close to the central strain.

The discrepancy between growth and consumption of lactose in whey for some of the strains prompted us to test whether the yeasts can assimilate lactose in the absence of other carbon sources. We precultured our lactose-utilizing strains in SC glucose and then transferred them to minimal medium (MM) supplemented with lactose only, and tracked growth of populations as the change in optical density in microscale (250 µL). The resulting growth curves revealed a variety of growth capacities, ranging from very fast growth (e.g., *A. mycotoxinivornas*, *S. (pseudo)intermedia*, and *P. laurentii*) to no detectable growth at all (e.g., *M. carribica*, *T. insectorum*, and *Y. galli*) (Fig. 4A). Moreover, some yeasts, including individual isolates of *M. carribica* and *Y. lipolytica*, displayed a long lag phase in liquid MM lactose, which may mean that the recorded doubling times do not reflect their maximal growth rates. Also, we cannot rule out the possibility that the absence of growth for some strains may reflect lag phases longer than the 72-h time frame of the experiment. Nonetheless, we found a substantial overlap between poor lactose consumption in liquid whey and poor growth in liquid MM lactose. Of the 17 species with an average lactose consumption of less than 10% in whey, 10 did not grow at all in liquid MM lactose, while the remaining species showed growth in only one or two out of several strains.

The ability to grow in MM lactose was next correlated with the expression of lactases, which cleave the lactose glucosidic bond. Many yeasts, including *Kluyveromyces*, are known to import lactose and break the lactose intracellularly (34), while others, such as *M. antarcticus*, secrete lactases and break the lactose glucosidic bond extracellularly (35) with the resultant monomers being imported and catabolized. To determine the degree and localization of lactase activities, we harvested end points (72 h) of the microscale MM lactose liquid cultures and separated cells from the extracellular liquid. We used a β-galactosidase activity assay to measure the lactase activity in both fractions. This revealed a range of different intra- and extracellular lactase activities (Fig. 4B). The isolates with no, or almost no, lactase activity (<1 absorbance at 420 nm) were all isolates that failed to grow on lactose, and all isolates with substantial lactose growth also displayed some lactase activity (>1 absorbance at ABS420). However, the capacity to break down lactose and convert it into yeast biomass (growth) was far from uniform. For example, the numerous *M. carribica* strains, which almost uniformly failed to grow in liquid MM lactose, displayed a range of (intracellular) lactase activity levels. In terms of lactase localization, the majority of the strains displayed preferentially intracellular breakdown of the glucosidic bond. However, interestingly, we note that many of the fastest lactose-growing isolates, including *M. antarcticus*, *U. sparsa*, and *A. mycotoxinivorans* and some, but not all, of the isolates of *S. pseudointermedia*, had both intra- and extracellular lactase activities. The results suggest that the lactose metabolic strategies vary substantially, both between and within species.

We hypothesized that the inability of some strains to grow in liquid MM lactose, despite having detectable lactase activities and despite growing on both whey and SC lactose agar, could be explained by the absence of externally supplied amino acids in MM. To clarify this, we streaked a subset of isolates that did not grow in liquid MM lactose (*M. carribica and M. guilliermondii*, *Y. lipolytica*, *Y. galli*, *C. tropicalis*, *T. insectorum*, and *K. ohmeri*) on MM agar plates supplemented with either glucose or lactose. We also included the auxotrophic *S. cerevisiae* lab strain BY4741, which cannot grow on MM due to the absence of uracil, histidine, leucine, and methionine, as a negative control. All strains, except *S*cBY4741, grew on MM glucose plates (Fig. 4C), strongly suggesting that they are prototrophs on a glucose medium. On MM lactose plates, we found *T. insectorum* to grow well, although it completely failed to grow in liquid MM lactose, showing that its lactose growth depends on the mode of cultivation. Differences in microbial growth on semi-solid medium versus in liquid medium have previously been shown to depend on surface attachment, nutrient accessibility, and oxygen diffusion (36, 37). Additionally, *M. carribica*, *M. guilliermondii*, *Y. lipolytica*, *Y. galli*, *C. tropicalis*, *T. insectorum*, and *K. ohmeri* formed microcolonies visible to the naked eye on MM lactose agar (Fig. 4D). These strains formed much larger colonies on SC lactose agar (Fig. S1), suggesting that their lactose growth depended on the media composition. Since the primary difference between MM and SC media is the presence of amino acids in the SC lactose medium, we hypothesize that these amino acids may serve as supplementary energy or carbon sources. Alternatively, their presence might facilitate lactose utilization by enabling the yeasts to more efficiently produce the proteins necessary for lactose uptake and metabolism.

During our re-streaking of isolates on separate SC lactose agar plates, we lost approximately 50% of the original 400 lactose-growing isolates identified on the colony arrays agar plates used for initial growth phenotyping of the whole collection (Fig. 2). Because some isolates were subsequently found to secrete lactases (Fig. 4B), we hypothesized that the loss of isolates could, at least partly, be explained by that they are not truly lactose assimilating, but rather that they grew in the initial arrays using extracellular lactases and monomeric breakdown products produced by adjacent colonies on the shared nutrient medium on the agar plates. Indeed, modeling shows nutrient and energy source diffusion through a shared agar medium to be fast, and substantially affect growth in colony arrays (38). To explore this hypothesis, we set up a feeding experiment, where one *A. mycotoxinivorans* strain with high levels of extracellular lactase activity was placed in the middle of an MM lactose agar plate and then surrounded by yeast isolates displaying poor or no growth when cultivated alone on this medium. *T. insectorum*, which autonomously produces intracellular lactases and grows on MM lactose agar, was used as a positive control. Indeed, the presence of *A. mycotoxinivorans* allowed slow but evident growth of all cells close to the feeder strain (Fig. 4E). While this communal aspect of yeast lactose growth bears some resemblance to natural microbial consortia, we note that it could serve as a confounding factor for the identification of lactose-growing strains in experimental setups where the isolates share the environment, such as agar plates. Likely, this also holds true for other carbon sources in our data set for which the degradation partially or completely occurs extracellularly, including plant biomass polymers such as cellulose and different hemicelluloses.

Overall, there seem to be multiple species and strains in our data set for which the capacity to grow on lactose is influenced by the cultivation system (liquid/solid) and media composition. We conclude that caution is needed when assigning a strain with the ability to grow on lactose.

### Basidiomycetous yeasts convert lactose into stored lipids

To function as a cell factory, a yeast strain must not only assimilate a substrate efficiently but also convert it into product(s) of industrial interest. A literature search showed that several of the identified yeasts, particularly the basidiomycetous species, have been reported as oleaginous yeasts, that is, they can produce and accumulate lipids to >20% of the dry cell weight.

**Fig 5:**
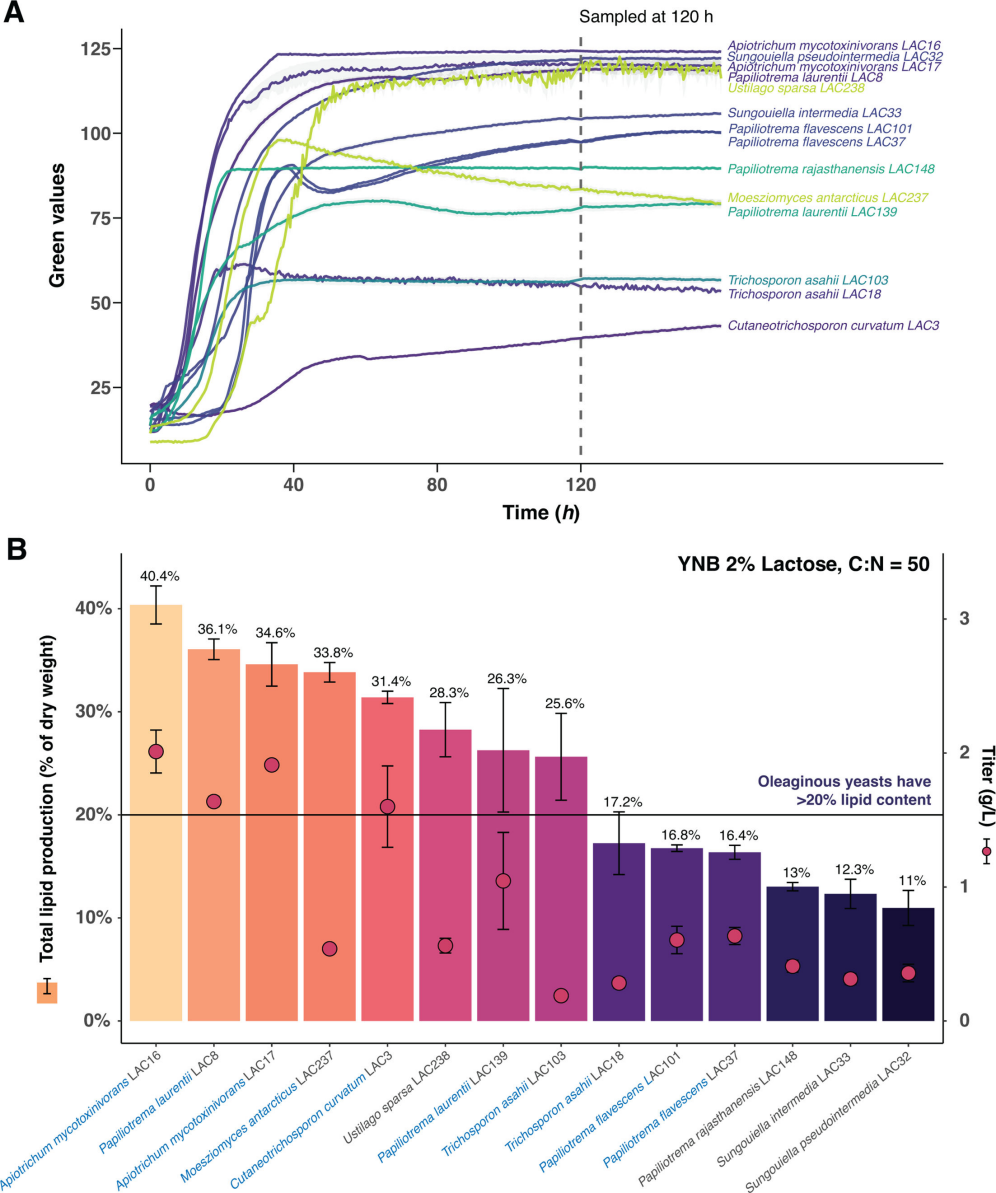
Yeasts converting lactose into lipids. Growth and lipid production in 14 selected yeast isolates growing in a carbon-rich, nitrogen-poor medium (C/N = 50) that maximizes lipid production. (**A**) Growth during microscale liquid cultivation. Each population size measure is an average of six replicates; fields indicate a standard error of the mean. (**B**) Total lipid yield in stationary phase cells, after 120 h of growth in liquid medium in shake-flasks. Lipid content is expressed as a percent of yeast dry weight (left *y*-axis) and lipid weight per liter of culture (right *y*-axis). Data shown represent an average of three independent cultures (*n* = 3). Error bars = standard deviation. All tested strains except *S. intermedia* and *S. pseudointermedia* belong to the *Basidiomycota* phyla. The cell dry weight values are reported in Table S3.

We therefore investigated the ability of 14 isolates, selected for their robust lactose-assimilation, to accumulate fatty acids from lactose under nitrogen-limited conditions (carbon-to-nitrogen [C/N] ratio 50), again using liquid MM lactose as it is completely devoid of additional carbon sources and gives full control over the C/N ratio. We first conducted 250 µL miniature cultures, tracking growth well into the stationary phase where most of the lipids are typically produced and stored (39), and identified 120 h as an appropriate time point for sampling (Fig. 5A). We then cultured the strains in 30 mL shake flask cultures (*n* = 3) for 120 h, extracted the total lipids from harvested and freeze-dried cells, and measured their lipid content relative to cell dry weight (40) (Fig. 5B). Over half (*n* = 8) of the 14 tested strains demonstrated substantial lipid production and accumulation (exceeding 20% of their total dry weight). The basidiomycetous strains previously recognized as oleaginous (strain names in blue in Fig. 5B) did indeed show high lipid content. To the best of our knowledge, lipid production from lactose has not been previously demonstrated for most of these species. This holds true also for *A. mycotoxinivorans*, the most prolific lipid producer found in our screen, which accumulated 40% of its cell dry weight in lipids. Of the species whose isolates failed to produce high lipid levels (<20% dry weight), none were previously known as oleaginous. We conclude that some of our best lactose-assimilating yeasts, including isolates of *A. mycotoxinivorans*, *P. laurentii*, *M. antarcticus*, and *C. curvatum*, have the capacity to effectively channel the lactose into lipids and thus demonstrate high cell factory potential.

## DISCUSSION

### Untargeted yeast bioprospecting uncovers isolates of potential industrial use

Tropical rainforests and other species-rich biomes can be found in Asia, Australia, Africa, Central and South America, Mexico, and many Pacific Islands. These biomes cover less than 20% of the Earth’s land area but may contain up to 50% of the planet’s biodiversity (8, 41). Numerous bioprospecting studies have been conducted in Central America, South America, and Asia (42–44), while Africa and Australasia remain underexplored sources of novel microorganisms, such as yeasts (8). As these biomes are under threat due to deforestation (45) and environmental changes, it is important to catalog their microbial biodiversity before it is lost.

We used an untargeted bioprospecting approach in the sense that we isolated yeasts on agar plates with a rich nutrient medium and glucose as carbon source, allowing the growth of a wide range of yeast species, while deselecting for bacteria by including three antibiotics in the medium. Admittedly, using a single isolation growth medium, we likely missed many species that require substantially different nutrients, pH, temperature, osmolarity, or the presence of other factors to grow, which could have been identified through a metagenomic study. However, the single isolation condition approach served an important purpose: all the isolated strains could subsequently be cultivated together on the same media plates and compared in an unbiased fashion. This makes it easy to extend the phenotypic characterization to encompass more environments of industrial interest. And while cultivating yeasts as colonies on a shared nutrient medium open for strain-strain interactions that can influence growth estimates, as demonstrated for lactose growth here, it probably better resembles the natural growth mode of most yeasts.

### Lactose growth capacity found in a diverse set of yeasts

Although we did not sample any lactose-rich environments, we found several yeast species from both the *Ascomycota* and *Basidiomycota* phyla that could grow on this carbon source. Lactases, the collective name of β-galactosidases and (less commonly) β-glucosidases active on lactose, are found in several glycoside hydrolase (GH) families that differ in structure, substrate specificity, catalytic mechanism, and optimal working conditions (such as pH and temperature), contributing to their functional diversity (27). Most likely, these enzymes initially evolved to degrade ubiquitous plant biomass-derived oligo- and disaccharides rather than lactose, which is a relatively scarce carbon source outside dairy farms. We hypothesize that upon extended exposure to lactose, yeasts that possess these enzymes with basic levels of activity towards lactose may evolve more efficient lactases relatively rapidly. Supporting this hypothesis, we found strains exhibiting lactase activity but no lactose growth, and substantial variability in the lactose-growth capacity among different strains of the same species. However, obtaining a comprehensive understanding of lactose metabolism across various species and strains would necessitate genome sequencing and detailed molecular characterization of their lactose catabolic traits, which falls outside the scope of this study.

Lactose metabolism has been studied in detail in *Kluyveromyces* yeasts, where lactose is imported across the cell membrane via a *LAC12*-encoded lactose permease and subsequently hydrolyzed by a *LAC4*-encoded lactase (a β-galactosidase from GH family 2) (46). The *LAC12* and *LAC4* genes are co-regulated and sit next to each other in the genome, forming a metabolic gene cluster (the *LAC* cluster) (47). In contrast, little is known about how lactose is hydrolyzed and taken up by most other yeasts. For example, some of the best lactose-growing yeasts in our data set, *A. mycotoinivorans*, *P. laurentii*, and *C. curvatum*, have not yet been thoroughly characterized. Interestingly, these fast-growing basidiomycetous yeasts exhibited both intra- and extracellular lactase activities, and the GH families of these enzymes remain unknown. Future research includes characterizing these lactases, which may possess different properties in terms of optimal pH and temperature, substrate specificity, and end-product inhibition, compared to lactases from *Kluyveromyces* (GH2) and *Aspergillus* (GH1) currently dominating in industrial production of lactose-free dairies.

Surprisingly, we did not isolate any lactose-assimilating *Kluyveromyces* strains in our collection. Instead, the most frequently sampled species were *S. intermedia* and its sister species *S. pseudointermedia*. They belong to the *Metschnikowia* family of ascomycetous yeasts, and are, to the best of our knowledge, the only species in this family that display robust lactose growth (48). Our previous work on *S. intermedia* shows that this species possesses both the conserved *LAC* cluster and an additional gene cluster, the *GALLAC* cluster, which is unique for *S. intermedia* and essential for its lactose growth (48). The exact phylogenetic relationship between *S. intermedia* and *S. pseudointermedia* has not yet been determined, and whether *S. pseudointermedia* also possesses a *GALLAC* cluster remains to be elucidated. We also isolated multiple strains of *M. carribica* and the closely related yeast *M. guilliermondii*. In contrast to *S. (pseudo)intermedia*, the isolated *Meyerozyma* strains did not display significant lactose consumption in whey nor robust growth in liquid MM lactose medium (although they grew on SC lactose agar plates). Genomic analysis of the type strains of both *M. carribica* and *M. guilliermondii* shows that they possess the conserved *LAC* cluster, confirming their genetic prerequisites for lactose metabolism (unpublished results). Follow-up studies will focus on elucidating the relationship between the ability to grow on lactose, the genetic setup, and the composition of the growth medium, both for these *Meyerozyma* strains and for other strains displaying inconsistent lactose growth within the collection.

### Basidiomycetous yeasts—cell factory potential for conversion of lactose into lipids

Our study identified several oleaginous basidiomycetous strains capable of lipid production when grown under nitrogen-limiting conditions with lactose as the sole carbon source. Given that these yeasts demonstrate efficient lactose consumption, it is highly likely that the lipids are produced through the conversion of lactose. Currently, yeast lipids are mostly produced from single mono- and disaccharides, hydrolysates, and glycerol, accounting for around 70% of the feedstocks used. In contrast, whey accounts for less than one percent of the feedstocks, although it is sizable, available, and cost-competitive (49). This underutilization of whey can be attributed, in part, to the limited metabolic capacity of known oleaginous yeasts to metabolize lactose. As such, our findings open new possibilities for using whey for lipid production in the future.

To date, over 160 yeast species have been described as having the ability to store more than 20% (wt/wt) of their dry weight as lipids, thereby termed oleaginous. Literature is dominated by *C. oleaginous*, *R. toruloides*, and *Y. lipolytica*, while many other oleaginous yeasts are still poorly characterized (49). Neither *C. oleaginous* nor *R. toruloides* were found in our screen, but we identified 10 strains of *Y. lipolytica*. However, they did not readily consume lactose in whey, nor did they grow well in MM lactose, consistent with literature stating that *Y. lipolytica* is a poor lactose-grower (50). Instead, we found several basidiomycetous, oleaginous yeasts, including *A. mycotoxinivorans* (formerly *Trichosporon mycotoxinivorans*), *P. laurentii*, *M. antarcticus*, and *C. curvatum* (formerly *Apiotrichum curvatum*), which all readily assimilated lactose in whey and produced high levels (25–40%) of lipids.

While we used MM lactose for lipid production, as this allowed us to set a defined, high C/N ratio to promote lipid biosynthesis and accumulation, we see no reason why cheese-whey could not serve as an effective substrate for these yeasts. In fact, optimizing the cultivation conditions regarding temperature, feeding strategy, and medium composition, including fine-tuning the C/N to perfectly fit the needs of individual species and strains, holds promise to significantly enhance lipid titers and yields (51). Moreover, genetic engineering can enable the construction of lipid-overproducing strains and/or strains in which β-oxidation and lipid turnover are abolished, ensuring that produced lipids are accumulated rather than consumed (52). We hope this study can help basidiomycetous yeasts gain the visibility they deserve for their superior lactose-to-lipid conversion capacities and cell factory potential.

### Conclusions and future perspectives

Bioprospecting is crucial for expanding our understanding of natural yeast biodiversity and identifying new yeasts with interesting ecological and biotechnological traits. We show that extensive strain collections from underexplored geographic regions can be valuable resources for identifying novel yeasts with potential as cell factories, paving the way for new and improved industrial applications. Our data set of lactose-assimilating yeasts revealed multiple species with exceptional lactose growth, previously unrecognized for this capability. We also found several strains that do not match known species particularly well, suggesting that they are new species. However, robust delineation and description of these novel yeasts will require comprehensive phenotypic profiling, DNA barcoding, and multi-locus phylogenetics (53). Finally, we demonstrate that some strains can produce substantial amounts of lipids from lactose, a trait that can be enhanced through further process optimization and strain development.

## MATERIALS AND METHODS

### Collection of source material and yeast isolation

#### Nigerian yeast collection

Different yeast source materials were collected from source environments and geographical regions across Nigeria (Fig. 1). Samples were collected in sterile plastic bags in the morning (16-20°C) and transported to the lab within 24 h. One unit of source material was mixed with nine units of sterile water to create a stock solution. Immediately after mixing, the solution was 10-fold diluted in sterile water. From the dilutions, 100 µL was spread on sterile Saccharomyces *Sensu Stricto* Enrichment Medium (SSEM) (54), containing yeast extract (3 g/L), malt extract (3 g/L), bacto peptone (5 g/L), glucose (10 g/L), and chloramphenicol (200 mg/L) and 2% agar. The plates were cultured at 25–28°C for 24–72 h because most yeasts are mesophilic (55). Single colonies were pure streaked, and their colony morphology was analyzed. Selected strains were transferred to SSEM agar in 96-well plates and transported to Sweden for storage. To keep only yeast strains, the final strain collection was further selected on chloramphenicol (200 mg/L), ampicillin (100 mg/L), and gentamycin (20 mg/L) and then long-term stored at −80°C in 20% (wt/vol) glycerol.

#### Lactose yeast collection

To isolate wild yeasts utilizing lactose as a sole carbon source, the collection of 1,996 strains was pinned to unbuffered SC medium composed of 0.14% Yeast Nitrogen Base (CYN2210, ForMedium), 0.5% NH4SO4, 0.077% Complete Supplement Mixture (CSM; DCS0019, ForMedium), 2.0% (wt/vol) glucose, and 0.6% (wt/vol) NaOH, with 2.0% (wt/vol) agar and 2% lactose. After 7 days of incubation at room temperature, lactose-growing colonies were streaked for single colonies on fresh SC lactose medium. Positive strains were streaked through single colony bottlenecks two to three times to ensure pure isolates. Strains were observed microscopically to confirm their identity. The strains were cultured in YPD (Yeast Peptone Dextrose) composed of 1.0% (wt/vol) Yeast extract, 2.0% (wt/vol) Peptone, and 2.0% (wt/vol) glucose overnight at 30°C and stored in 20% glycerol at −80°C in 96-well microtiter plates.

#### Species identification

The ITS region was sequenced using the primer sets (ITS1, 5′-TCCGTAGGTGAACCTGCGG-3′) and (ITS4, 5′-TCCTCCGCTTATTGATATGC-3′). Species identification was performed by NCBI BLAST against the standard Nucleotide collection and the ITS Fungi type and reference material database. The top hits from both databases were used to determine species identities, and the percentage identities were recorded in Table S1.

### Yeast growth media and phenotyping conditions

#### Storage and recovery of strains

All yeast strains were stored at −80°C in 20% glycerol. The yeast collection was recovered and distributed in arrays on solid agar plates by robotic pinning (ROTOR HDA, Singer Instruments, UK). The solid medium Singer Plus plates (Singer Instruments, UK) were cast on a leveled surface with 50 mL agar medium added and air-dried at room temperature for 2 days before use. Frozen glycerol stocks of yeast strains were recovered on YPD medium. Except during the recovery of frozen glycerol stocks, yeast strains were pre-cultivated on SC medium pH buffered to 5.8 with 1.0% (wt/vol) succinic acid, and 0.6% (wt/vol) NaOH, with 2.0% (wt/vol) agar to solidify the medium. In nitrogen-limiting environments, the background medium was modified to avoid confounding growth on stored nitrogen by replacing CSM with 20 mg/L uracil and reducing the (NH_4_)_2_SO_4_ concentration to 30 mg Nitrogen/L.

#### Growth phenotyping media

Carbon environments comprised SC medium, with various concentrations or other carbon sources replacing 2.0% glucose. Stress environments were prepared by supplementing SC medium with various compounds while keeping glucose as the sole carbon source at 2.0%. Nitrogen-limiting environments were prepared using a modified SC medium with 20 mg/L uracil instead of CSM and supplemented with various nitrogen sources to 30 mg Nitrogen/L. Carbon environments were prepared using other sugars or concentrations instead of glucose. Liquid cultures and, in a few cases, solid media environments were composed of MM medium and contained 2.0% agar (wt/vol), 20 g/L lactose or glucose, 5 g/L ammonium sulfate, 3 g/L potassium phosphate, 1 g/L magnesium sulfate, vitamins, and trace metals as described previously (56), with pH adjusted to 5.0 using 2 M KOH.

#### Cheese whey preparation

Cheese whey was prepared fresh using fresh non-homogenized whole milk with a fat content of 3.8–4.5%. A quarter of a rennet tablet (Fromase 50, DSM Food Specialities) was dissolved in 50 mL H_2_O. Separately, 7.5 mL citric acid powder was dissolved in 50 mL H_2_O. A volume of 4.5 L milk was added to a pan, and the citric acid solution was carefully spread in the milk with a slotted spoon. The solution was slowly heated to 32°C and removed from the heat source. The rennet solution was added, and the solution was left undisturbed under a lid for 20 min. The cheese curds were cut, and the solution was heated to 40°C. The cheese whey was separated from the curds and filter-sterilized to remove any potential contaminants.

### Growth phenotyping in high-throughput

#### Solid media growth phenotyping

Yeast strains were arranged in colony arrays and individually tracked for growth using the Scan-o-matic system (24) version 2.2 (https://github.com/Scan-o-Matic/scanomatic/releases/tag/v2.2). Solid media plates were secured in position by an acrylic fixture, left undisturbed without lids for 48 h in high-resolution desktop scanners (Epson Perfection V800 PHOTO scanners, Epson Corporation, UK) kept inside dark, humid, and temperature-controlled (30°C) thermostatic cabinets. Colony growth on the plates was recorded by sequential transmissive scanning at 600 dpi resolution, with four plates per scanner, during 20-min intervals. Pixel intensity normalization and standardization were performed across different scanners and experimental runs using a transmissive grayscale calibration strip. Each scanned plate had a virtual grid placed across it, with intersections at the center of each colony. Colonies and the surrounding agar medium were segmented, and local background was separated from colony pixel intensities. A pre-defined *S. cerevisiae* calibration function, based on spectroscopic and flow cytometer measurements (24), was used to convert colony pixel intensities to cell numbers. Population size growth curves were obtained, and growth rates were identified at the steepest slope using a local regression. The growth curves were processed to remove noise, and erroneous curves were manually inspected and excluded from the analysis. Growth phenotypes were extracted as numerical values from all growth curves that passed the quality requirements (Table S2).

#### Liquid media growth phenotyping

A subset of strains capable of growing on lactose as a carbon source were further analyzed in liquid media. Strains were precultured for 72 h in 96-well microtiter plates. In Growth Profiler 96-well plates, 250 µL fresh media was inoculated with a 5 µL saturated starter culture. The plates were mounted in the Growth Profiler 960 (Enzyscreen), and the cultures were imaged at 30 min intervals, as controlled by the Growth Profiler Control v6.1.0. The increase in culture turbidity was detected by the software GP960Viewer and reported as green values. Growth curves were analyzed using PRECOG (57), and the single numeric growth parameters generation time, lag, and yield were extracted.

### Detection of metabolites and bioproducts

#### β-Galactosidase activity

β-Galactosidase activity was detected using the Yeast β-Galactosidase Assay Kit (75768, Thermo Scientific) following the microplate protocol provided by the manufacturer. After recording the final time point, 100 µL of the liquid cultures was transferred to new 96-well plates. The plates were centrifuged at 5,000 × *g* for 10 min to pellet the cells. The intracellular (INT) and extracellular (EXT) activities were separated by transferring 80 µL of the supernatant from each well to a new plate (EXT), while the remaining cell pellets were washed with 180 µL of distilled water in the original plate (INT). Both plates were centrifuged again at 5,000 × *g* for 10 min. For the INT plate, the supernatant was discarded, and 70 µL of distilled water was added to the pellets. For the EXT plate, 70 µL of the supernatant was transferred to a new plate (EXT2). Next, 70 µL of a 1:1 mixture of Y-PER reagent and assay buffer was added to each well of both plates (INT and EXT2). The plates were incubated at 37°C with shaking at 400 rpm. After 15 min, the reactions were stopped by adding 56 µL of stop solution to each well. The plates were then mixed for 10 s at 400 rpm. The absorbance was measured using a plate reader (FLUOstar Omega) at 420 nm.

#### High-performance liquid chromatography

Yeast cultures were centrifuged in their respective wells of the 96-well GrowthProfiler plates at 5,000 × *g* for 10 min. For each sample (*n* = 1), 100 µL of the supernatant was 10-fold diluted; the cells were removed by passing the 1 mL diluted samples through a syringe filter (0.22 µm polyethersulfone). The processed samples were transferred to HPLC glass vials. The analysis aimed to detect media components and metabolites using an Aminex HPX-87H carbohydrate analysis column (Bio-Rad Laboratories). The injection volume was 5 µL, and HPLC column was maintained at 80°C, and 5 mM H_2_SO_4_ served as the mobile phase at a constant flow rate of 0.8 mL/min. Chromatogram peaks were treated and integrated using the Chromeleon v6.8 software (Dionex).

#### Total lipid extraction

The lipid content was determined from dry cell weight using a protocol adapted from reference (40). Yeast strains were cultivated in 2% lactose YNB medium without added amino acids and with a C/N ratio of 50, and the cultures were incubated at 30°C for 120 h. In 250 mL shake flasks, strains were inoculated to an OD_600_ of 0.1 in 30 mL media in triplicate. After incubation, cells were harvested by centrifugation at 3,000 × *g* for 5 min, washed two times in double-distilled sterile water, and freeze-dried (Labconco 7753011) for 24 h. The dry weight of the cells was measured. Cell disruption was achieved by adding 1.5 mL of 2M HCl and incubating at 90°C and 700 rpm for 1 h. Subsequently, each sample received 3 mL of a 1:2 (vol/vol) chloroform-methanol solution and was mixed at 1,000 rpm at 25°C for 10 min. After that, 1 mL chloroform and 0.8 mL 0.88% (wt/vol) KCl was added and the samples were mixed at 1,000 rpm at 25°C for 5 min, followed by centrifugation at 3,000 × *g* for 10 min. The lower chloroform phase was transferred to pre-weighed tubes and centrifuged again. Any traces of an upper aqueous phase in the chloroform samples were removed. Solvents were evaporated by flushing N_2_ gas for 48 h using an evaporator. Finally, total lipid extracts were weighed, and lipid content was calculated by dividing the lipid weight by the cell dry weight.

## Data Availability

Strains have been collected and handled in accordance with the Nagoya Protocol on Access to Genetic Resources and the Fair and Equitable Sharing of Benefits Arising from their Utilization to the Convention on Biological Diversity (https://www.cbd.int/ABS). They remain the property of Nigeria which regulates access through its Nagoya protocol node, through which access can be negotiated. Sequences can be accessed in table S1 and on Genbank accession PQ758410-PQ758587 (https://www.ncbi.nlm.nih.gov/nuccore/?term=PQ758410:PQ758587[accn]).

## SUPPLEMENTAL MATERIAL

Figure S1 aem.01615-24-s0001.eps Colony sizes of isolates streaked on SC agar medium with lactose 2%.

Figure S2 aem.01615-24-s0002.eps Conversion of cheese whey into yeast biomass and metabolites.

Table S1 aem.01615-24-s0003.txt Information on individual lactose-assimilating strains and their sources of isolation.

Table S2 aem.01615-24-s0004.txt Growth phenotypes.

Table S3 aem.01615-24-s0005.txt Cell dry-weight values.

ASM does not own the copyrights to Supplemental Material that may be linked to, or accessed through, an article. The authors have granted ASM a non-exclusive, world-wide license to publish the Supplemental Material files. Please contact the corresponding author directly for reuse.
